# CRediT for authors of articles published in the *Journal of the Medical Library Association*

**DOI:** 10.5195/jmla.2021.1294

**Published:** 2021-07-01

**Authors:** Kristine M. Alpi, Katherine G. Akers

**Affiliations:** 1 krisalpi@gmail.com, University Librarian, Oregon Health & Science University Library; 2 jmla@journals.pitt.edu, Editor-in-Chief, Journal of the Medical Library Association

## Abstract

To help ensure that authors of articles published in the *Journal of the Medical Library Association* (*JMLA*) receive appropriate recognition for their contributions and to make individual author roles more transparent to readers, *JMLA* articles will begin including Author Contribution statements using the Contributor Role Taxonomy.

The collaborative nature of the intellectual work of health sciences librarians is increasingly evidenced by coauthored journal articles, including those published in the *Journal of the Medical Library Association* (*JMLA*) [1]. Librarians also work diligently to negotiate coauthorship on manuscripts resulting from collaborations with researchers outside of librarianship [2, 3], particularly in the realm of systematic reviews [4]. However, direct conversations about coauthorship can be difficult for a myriad of reasons [5]. Also, although the order of authors on published articles often conveys information about the significance of their individual contributions, a lack of transparency about how each author contributed to a project can be at best confusing and at worst serve to mask unethical practices such as “honorary authorship” [6].

Guidance on determining authorship is provided by the International Committee of Medical Journal Editors (ICMJE) [7]. However, ICMJE does not provide standardized language for describing individual author contributions. Rather, the Contributor Role Taxonomy (CRediT) is the preeminent approach to describing fourteen different contributor roles in scientific scholarly output (Table 1) [8]. CRediT emerged following a 2012 collaborative workshop led by Harvard University and the Wellcome Trust with input from researchers, ICMJE, and publishers and facilitation by the Consortia Advancing Standards in Research Administration (CASRAI) and National Information Standards Organization. This taxonomy has been adopted by many major journals and journal publishers, including Elsevier's *Journal of Academic Librarianship*.

**Table 1:** Fourteen contributor roles defined by CRediT [8]

Contributor role	Definition
Conceptualization	Ideas; formulation or evolution of overarching research goals and aims.
Data curation	Management activities to annotate (produce metadata), scrub data and maintain research data (including software code, where it is necessary for interpreting the data itself) for initial use and later re-use.
Formal analysis	Application of statistical, mathematical, computational, or other formal techniques to analyze or synthesize study data.
Funding acquisition	Acquisition of the financial support for the project leading to this publication.
Investigation	Conducting a research and investigation process, specifically performing the experiments, or data/evidence collection.
Methodology	Development or design of methodology; creation of models.
Project administration	Management and coordination responsibility for the research activity planning and execution.
Resources	Provision of study materials, reagents, materials, patients, laboratory samples, animals, instrumentation, computing resources, or other analysis tools.
Software	Programming, software development; designing computer programs; implementation of the computer code and supporting algorithms; testing of existing code components.
Supervision	Oversight and leadership responsibility for the research activity planning and execution, including mentorship external to the core team.
Validation	Verification, whether as a part of the activity or separate, of the overall replication/reproducibility of results/experiments and other research outputs.
Visualization	Preparation, creation, or presentation of the published work, specifically visualization/data presentation.
Writing—original draft	Preparation, creation, or presentation of the published work, specifically writing the initial draft (including substantive translation).
Writing—review & editing	Preparation, creation and/or presentation of the published work by those from the original research group, specifically critical review, commentary or revision—including pre- or post-publication stages.

The use of CRediT to describe author contributions in published articles confers multiple benefits to the scholarly community [8–10]. Together with ICMJE guidelines, CRediT can be used to facilitate conversations and help determine who merits authorship within collaborative teams. The broad range of CRediT-defined contributor roles, which span the research lifecycle, recognizes the diverse skills and resources that different researchers bring to a team, potentially leading to the greater inclusion of individuals as coauthors. Knowing which author was responsible for which aspect of a project can help researchers seek out new collaborators and aid decision-making by grant funders and tenure and promotion committees. The delineation of roles played by specific authors can inform scientometric or “science of science” research and be used to develop new metrics of individual research productivity and impact. Furthermore, as health sciences library and information professionals, applying contributor roles would allow us to acknowledge, analyze, and evaluate our own contributions to scholarship and contribute to the training of future authors.

To help realize these benefits, as of July 1, 2021, Knowledge Synthesis, Original Investigations, Case Reports, Special Papers, and Virtual Projects manuscripts submitted to *JMLA* must contain an Author Contribution statement that uses CRediT to specify the role(s) played by each author. An example Author Contributions statement is as follows:

Brittany Eagleton: Methodology; investigation; writing—review and editing. John Tramos: Conceptualization; methodology; investigation; software; data curation; formal analysis; visualization; writing—original draft. Lawrence Kanumba: Conceptualization; project administration; formal analysis; writing—original draft; Changying Wang: investigation; formal analysis; visualization; writing—review and editing.

We recognize that CRediT does not currently describe all roles that librarians play in contributing to publications. One effort to expand the taxonomy that includes health sciences librarians is the Contributor Role Ontology, an open source, community-developed ontology with over fifty terms through which persons or organizations can be credited for their contributions to research-related artifacts [11]. However, as a first step, adopting CRediT will help ensure that authors of *JMLA* articles receive appropriate recognition for their contributions and will serve to make individual author roles more transparent to readers. *JMLA* welcomes your thoughts and feedback on this or any other topic, which can be sent to the editor-in-chief at jmla@journals.pitt.edu or submitted through JMLA's virtual suggestion box (https://www.mlanet.org/p/su/rd/survey=dd8fcbda-649f-11eb-8b4f-bc764e103913).
